# PCA-induced respiratory depression simulating stroke following endoluminal repair of abdominal aortic aneurysm: a case report

**DOI:** 10.1186/1752-1947-1-45

**Published:** 2007-07-10

**Authors:** Javed Ahmad, Richard Riley, Kishore Sieunarine

**Affiliations:** 1 Departments of Vascular Surgery and Anaesthesia, Royal Perth Hospital, Perth, WA 6000, Australia

## Abstract

**Aim:**

To report a case of severe respiratory depression with PCA fentanyl use simulating stroke in a patient who underwent routine elective endoluminal graft repair for abdominal aortic aneurysm (AAA)

**Case presentation:**

A 78-year-old obese lady underwent routine endoluminal graft repair for AAA that was progressively increasing in size. Following an uneventful operation postoperative analgesia was managed with a patient-controlled analgesia (PCA) device with fentanyl. On the morning following operation the patient was found to be unusually drowsy and unresponsive to stimuli. Her GCS level was 11 with plantars upgoing bilaterally. A provisional diagnosis of stroke was made. Urgent transfer to a high-dependency unit (HDU) was arranged and she was given ventilatory support with a BiPap device. CT was performed and found to be normal. Arterial blood gas (ABG) analysis showed respiratory acidosis with PaCO_2 _81 mmHg, PaO_2 _140 mmHg, pH 7.17 and base excess -2 mmol/l. A total dose of 600 mcg of fentanyl was self-administered in the 16 hours following emergence from general anaesthesia. Naloxone was given with good effect. There was an increase in the creatinine level from 90 μmol/L preoperatively to 167 μmol/L on the first postoperative day. The patient remained on BiPap for two days that resulted in marked improvement in gas exchange. Recovery was complete.

## Background

Endoluminal repair for abdominal aortic aneurysms (AAA) has become an established technique for patient undergoing elective surgery. Repair is usually achieved with small groin incisions that may be managed with less aggressive analgesia regimens than those reserved for open repair [1].

## Case presentation

A 78-year-old woman was admitted for elective repair of a 5.5 cm AAA. She had past medical history of osteoarthritis and had undergone bilateral mastectomy in 1995. She had a transient ischaemic attack (TIA) 2 months prior to surgery with no carotid stenosis.

She underwent endoluminal graft repair for AAA performed under general anaesthesia. Bilateral inguinal incisions were made and femoral arteries were the main access vessels on both sides. She had an uneventful surgery. The aorta was the only artery affected by the aneurysm. The Talent^® ^(Medtronic Corporation, California) aorto-bi-iliac-bifurcated graft device was used. The aneurysm was successfully excluded. The operation was completed in 2.5 h. There were no complications at the time of the procedure and no additional procedures were necessary. During the operation there was 350 mL of blood loss and 3000 units of heparin was used with 2 mg of morphine. She had an uneventful recovery. She was transferred to the surgical ward from the post-anaesthesia care unit.

Next morning she was noted to be unusually drowsy and difficult to rouse. Neurological examination revealed a GCS of 11 (E3 + M4 + V4) with flaccid limbs and bilateral upgoing plantar reflexes(Preoperative plantar reflexes were normal). A stroke was suspected and a CT head performed at this time was normal. The blood pressure was 170/70 mmHg and the respiratory rate was 12 per minute. Arterial blood gases showed respiratory acidosis (pH = 7.17, PCO_2 _= 81 mmHg, PO_2 _= 140 mmHg, HCO_3 _= 29 mmol/l, base excess = -2 mmol/l, O_2 _saturation = 98%, anion gap 12). She was immediately transferred to the High-Dependency Unit (HDU) where BiPap (ventilatory support) was commenced. Naloxone 200 μg was given. Preoperative and post-awaken arterial blood gases without respiratory support were not available.

The blood pressure remained stable throughout this period (BP = 170/70 mmHg, pulse = 75/min, CVP 7 cm H_2_O). ECG showed sinus rhythm. On examination the lungs were clear, the abdomen was soft and wound drainage was not significant. Renal function was normal preoperatively (creatinine = 90 μmol/l) but became elevated in the post operative period. (creatinine = 167 μmo/l). There was an episode of rapid atrial fibrillation (AF) on day 2 postoperatively which resolved after IV amiodarone. There was a small troponin rise to 0.9 μg/l.

She remained in HDU for two days where she showed progressive improvement with ventilatory support. On close questioning the patient revealed a history of disturbed sleep with multiple awakenings, snoring and feeling unrefreshed in the morning. She would fall asleep frequently during the day. A provisional diagnosis of Obstructive Sleep Apnoea (OSA) was made which was confirmed with sleep studies 7 months later.

The patient used patient controlled analgesia(PCA)with fentanyl for postoperative pain relief with a total dose of 600 μg in 16 hours. She received an initial bolus of 1 mg morphine intravenously after the operation after which PCA machine was used. The PCA machine was programmedto deliver 20 μg bolus of fentanyl with a five minute lockout time interval. She improved with ventilatory support over the following two days, weaned to Venturi face mask and then discharged on room air. PCA pumps are used when the duration of surgery is long or the wound is large or there is significant fentanyl use in the recovery room. This is our practice in Royal Perth Hospital. This patient had large inguinal incisions. Moreover 600 mcg of fentanyl is not unsual consumption in 16 hours.

This dose was evenly distributed over 16 hours. Postoperatively a small lymphocoele developed in left groin that was treated conservatively. Postoperative CT angiogram showed no signs of leak. There was no follow up CT Scan or MRI to exclude stroke as it was not indicated clinically.

## Discussion

Based on the Charles Dickens' character Joe, the fat boy in "The Posthumous papers of the Pickwick Club", Osler and later Burwell applied the name "Pickwickian Syndrome" to the combination of obesity, hypersomnolence, and the signs of chronic alveolar hypoventilation. Apnoea, both obstructive and central, had also been noted by bedside observation during sleep as early as 1877. Studies have shown that obstructive sleep apnoea is a common disorder that represents a huge public health problem [2,3].

We report a 78 year old lady with suspected OSA who developed severe respiratory depression following a successful endoluminal graft repair for AAA which is a routine procedure. She used PCA with fentanyl for postoperative pain and she had slightly impaired renal functions (creatinine 167 micromol/l). Her body weight was 107 kg and height was 1.73 metres, body mass index = 35.7 kg/m^2^. She was a non-smoker and had no history of COPD. A PCA with fentanyl (programmed with bolus size 20 μg, 5-minute lockout interval, no background infusion) was used every hour on the postoperative night to the extent that by next morning she received a total of 600 μg of fentanyl (16 hours after operation). As she had slightly impaired renal function (creatinine 167 μmol/l), this was probably an added factor in decreasing excretion of fentanyl from the body resulting in respiratory depression in a patient with OSA. She was so drowsy and unresponsive that a CT scan was performed to rule out stroke. She had a full recovery with ventilatory support with Bipap for two days.

While it is not unexpected that respiratory depression might be seen in patients with obesity and OSA, this case was remarkable for an unusual presentation. OSA is a major health problem. A large prevalence study of United States employees found that undiagnosed sleep-disordered breathing is prevalent and has a wide range of severity in middle-aged women and men [4]. In this study, which was done in 1993, 9.1% of men and 4% of women had apnoea/hypopnoea indices of 15 or more events per hour. It is estimated that, in the United States alone, more than 3 million men and 1.5 million women meet at least one definition of OSA (apnoea/hypopnoea index of five or more plus a complaint of daytime sleepiness). Sleep apnoea encompasses a number of different clinical problems. In OSA, the most common form of sleep apnoea, episodes of apnoea occur during sleep as a result of airway obstruction at the level of the pharynx [4]. The preponderance of evidence indicates that the pharynx is abnormal in size and/or collapsibility in patients with OSA.

The cardinal manifestations of OSA syndrome are stentorian snoring and severe sleepiness. OSA is treated by surgical and nonsurgical means. Nonsurgical treatment includes weight loss, restriction of body position during sleep, avoidance of alcohol and upper airway mucosal irritants as well as selected drugs (nasal decongestants, steroids). Surgical treatment includes tracheostomy, tonsillectomy, adenoidectomy, nasal surgery and uvulo-palato-pharyngoplasty. It also appears that clinicians are recognising OSA in their patients with increasing frequency. In the USA there was a 12-fold increase in the annual number of patients diagnosed with sleep apnoea between 1990 and 1998. Despite the widespread prevalence of this problem, it seems that many case reports of patients with OSA are not reported [5-7].

The PCA technique has been used for over 20 years and has an impressive safety record. However, in Royal Perth Hospital in 1988, a potentially lethal complication was reported with the development of prolonged apnoea in an otherwise healthy fifteen-year-old male patient following appendicectomy. He recovered completely with brief ventilatory support and intravenous naloxone [8].

There is one fatal case reported due to respiratory arrest following excessive use of PCA. Two other cases of PCA-related respiratory arrest have been reported [9-11]. Patients with pre-existing medical conditions (such as OSA, renal or cardio-respiratory disease) that will influence their postoperative analgesic regimen need to be managed in an appropriate setting (such as HDU) so that adverse effects can be detected early. In addition, surgical teams need to be alert for this potential, rare complication in patients who are managed postoperatively in the general ward as prevention is the best solution.

## Conclusion

This case illustrates that use of analgesia (PCA) can produce severe respiratory depression in a patient following routine endoluminal graft repair of AAA. For selected patients, PCA use may not be warranted following endoluminal surgery.

## Competing interests

The author(s) declare that they have no competing interests.

## Authors' contributions

RR helped in rewriting and proof reading. KS helped in rewriting and proof reading.
